# Concurrent tissue and circulating tumor DNA analysis in renal cell carcinoma: insights from a multimodal database

**DOI:** 10.1093/oncolo/oyag123

**Published:** 2026-04-03

**Authors:** Chinmay T Jani, Elizabeth Tran, Ellen Jaeger, Jianjie Dong, Nabil Husni, Melissa C Stoppler, Adam J Hockenberry, Jacob Mercer, Sumanta Pal, Neeraj Agarwal, Toni K Choueiri, Brent Rose, Aditya Bagrodia, Rana R McKay

**Affiliations:** University of Miami/Sylvester Comprehensive Cancer Center, Miami, FL, 33136, United States; University of California San Diego, La Jolla, CA, 92037, United States; Tempus AI, Inc., Chicago, IL, 60654, United States; Scripps Institute, San Diego, La Jolla, CA, 92037, United States; Tempus AI, Inc., Chicago, IL, 60654, United States; Tempus AI, Inc., Chicago, IL, 60654, United States; Tempus AI, Inc., Chicago, IL, 60654, United States; Tempus AI, Inc., Chicago, IL, 60654, United States; City of Hope, Duarte, CA, 91010, United States; Huntsman Cancer Center, Salt Lake City, UT, 84112, United States; Dana-Farber Cancer Institute, Boston, MA, 02215, United States; University of California San Diego, La Jolla, CA, 92037, United States; University of California San Diego, La Jolla, CA, 92037, United States; Dana-Farber Cancer Institute, Boston, MA, 02215, United States

**Keywords:** kidney cancer, RCC, ctDNA, tissue NGS

## Abstract

**Introduction:**

Circulating tumor DNA (ctDNA) sequencing complements tissue-based next-generation sequencing (NGS), offering noninvasive and serial testing. We explore the mutational landscape of renal cell carcinoma (RCC) using matched tissue and ctDNA data to assess complementarity and clinical significance of molecular alterations.

**Methods:**

From the Tempus multimodal database, we retrospectively analyzed de-identified data from patients with RCC with concurrent tissue (Tempus xT) and ctDNA testing (Tempus xF). Patients with xT and xF matched samples (collected ±90 days of one another) were included. We evaluated socio-demographic and clinical characteristics and selected pathogenic somatic short variants (PSSVs) and copy number variants (CNV). Analyses were restricted to the 104 genes shared by all assays.

**Results:**

Among 392 patients, 66% (*n* = 259) had metastatic disease. The median time from tissue to blood collection was 21 days. The most common tissue sites were kidney (49%, *n* = 189) and bone (11%, *n* = 43). Frequently altered tissue-tested genes were: *VHL* (59%), *PBRM1* (32%), and *SETD2* (23%). Most frequently altered genes in ctDNA were TP53 (23%), VHL (18%), BAP1 (6%), and PBRM1 (5%); notably, 176 patients did not have any pathogenic or likely pathogenic variants detected in the 104 genes analyzed. Complementary ctDNA and tissue testing detected 6% more alterations than tissue testing alone, with greater concordance in metastatic cases.

**Conclusion:**

ctDNA testing offers complementary insights to tissue NGS in RCC, particularly in metastatic disease, suggesting the potential utility of ctDNA in advanced RCC. Longitudinal analysis may enhance delineation of biomarkers of response and resistance at mutation and ctDNA fraction levels.

Implications for Practice Circulating tumor DNA (ctDNA) can provide actionable insights in advanced renal cell carcinoma (RCC), complementing tissue-based next-generation sequencing (NGS). In this analysis of matched tissue and ctDNA from a large RCC cohort, ctDNA identified clinically relevant alterations, particularly in metastatic disease, and captured mutations not detected by tissue alone. These findings support the use of ctDNA as a noninvasive tool to enhance molecular profiling, guide targeted and enable real-time monitoring of treatment response and resistance. Incorporating ctDNA into routine practice may improve precision oncology approaches for patients with advanced RCC.

## Introduction

Next-generation sequencing (NGS) of circulating tumor DNA (ctDNA) has emerged as a powerful complement to tissue NGS, offering a noninvasive and serially conductible test.^1^ ctDNA analysis has become an invaluable tool in the landscape of oncology, facilitating real-time monitoring of tumor dynamics and therapeutic response across various solid cancers.^2^^,^^3^ The ability of ctDNA to capture tumor heterogeneity, identify genomic alterations to guide therapy selection, and monitor the emergence of resistance alterations, emphasizes the role of liquid biopsies as a crucial tool for precision oncology.^4^^,^^5^ In the localized setting, ctDNA holds the promise to be a marker of minimal residual disease (MRD).^6^ Recent research has highlighted the utility of ctDNA in various solid tumors, including lung, colorectal, and breast cancers, where it has shown high concordance with tissue biopsies.^7–9^ For instance, in non-small cell lung cancer (NSCLC), ctDNA profiling has been employed to track the dynamics of *EGFR* mutations during targeted therapy.^7^^,^^8^ Similarly, in colorectal cancer, ctDNA analysis has been used to detect *KRAS* mutations and predict prognosis and outcomes.^9^

Renal cell carcinoma (RCC), a heterogeneous malignancy with a complex genomic landscape, presents unique challenges and opportunities for ctDNA analysis.^10^ RCC often exhibits spatial and temporal molecular heterogeneity, which complicates treatment strategies and suggests the benefit of comprehensive molecular profiling.^11^^,^^12^ Despite challenges of low ctDNA shedding rates and technical detection limitations in RCC patients, ctDNA can provide a holistic view of the tumor’s genetic makeup.^10^^,^^13–16^ Recent studies have explored the concordance between ctDNA and tissue DNA in RCC, demonstrating that ctDNA can accurately reflect key genetic alterations found in tissue samples.^17^^,^^18^ These findings highlight the potential of ctDNA to serve as a potential surrogate for tissue biopsies.

In this study, we built on these foundational insights by exploring the mutational landscape of RCC patients through comprehensive profiling of mutations in ctDNA and matched tissue samples. Utilizing a multimodal real-world database, we sought to elucidate the agreement and clinical significance of molecular alterations detected in both circulating and tissue-derived DNA. Our analysis leveraged advanced sequencing technologies and longitudinal data to capture the heterogeneity of RCC at a molecular level. By comparing genomic alterations in ctDNA with those in tissue biopsies, we provide a deeper understanding of tumor molecular alterations, supporting the potential for ctDNA to guide personalized treatment strategies in RCC.

## Methodology

### Data collection

Patients with RCC who had undergone concurrent testing with both tissue NGS (Tempus xT) and ctDNA NGS (Tempus xF/xF+) were identified. The Tempus xT panel analyzes 596-648 genes, while the Tempus xF/xF+ panels assess 105-523 genes.^19^^,^^20^ Only the 104 genes common to all solid-tissue and ctDNA panels were reported (Table S1). Inclusion criteria for the study was a diagnosis of RCC, and the availability of both tissue and ctDNA NGS results where the matched tissue/ctDNA sample collection occurred within a 90 day window of one another—this temporal proximity ensured that the samples were representative of the same disease state, minimizing variability due to tumor evolution over time. Patients with clear cell, chromophobe, papillary, medullary, collecting duct, translocation, and not otherwise specified (NOS) histologies were included, with all other histologies excluded.

### Data analysis

We extracted and analyzed available socio-demographic information (age, sex, race/ethnicity) and clinical characteristics (presence or absence of metastases at time of sample collection, tissue sample location and previous treatments) from the Tempus multimodal database for the set of patients meeting our inclusion/exclusion criteria. For genetic variants, the primary focus was on pathogenic somatic short variants (PSSVs) for both solid-tissue and ctDNA testing, as well as copy number variants (CNVs), including amplifications (copy number of 8 or greater) and deletions (2 copy number losses) specifically in solid tissue testing. Tempus xT utilizes a normal match component to determine the source of the variant: somatic or potential germline (63 genes approved for potential germline reporting, Table S2). The patient’s normal tissue was derived from the plasma and buffy coat from Streck tubes, isolated and used for sequencing. The prevalence of germline alterations was calculated only from patients with tumor-normal match testing (*n* = 355, 91% of cohort).

### Comparisons between solid-tissue and ctDNA results

Alterations between tissue and ctDNA assays were evaluated for the 104 genes common to all solid-tissue and ctDNA panels, with a focus on clinically relevant genes: *VHL*, *PBRM1*, *TP53*, *TERT*, *BAP1*, *ARID1A*, *BRCA2*, *TSC1*, *MTOR*, and *ATM*. Analyses were further restricted to PSSVs. Note that some “solid-tissue only” variants may be due to specific differences in exonic and intronic panel coverage between the xT and xF assays (eg, certain exons in *PBRM1*, *TERT*, *TSC1*, *mTOR*, and *ATM* are not specifically targeted in the xF panel). The complementary detection of individual genes was assessed by categorizing variants and assessing the proportion of patients harboring an alteration in the gene of interest that was detected only in tissue, only in ctDNA samples, or in both tissue and ctDNA samples divided by the total number of patients in the study.

### Clonal hematopoiesis (CH) variant analysis

To identify CH variants, somatic variants initially detected only in the ctDNA (xF) assay were cross-referenced with data from matched-normal buffy coat samples sequenced via the Tempus xT assay for the same patients. A variant was classified as suspected CH if it was detected in the matched-normal sample with a variant allele frequency (VAF) 1.5× greater than that in the tumor sample.

### Subgroup analysis

To understand the impact of metastases on detection differences between assays, we stratified patients based on the presence or absence of metastatic disease prior to both tissue and liquid biopsy collection (13 patients were excluded from this analysis due to conflicting or unknown timing of metastatic diagnoses). Variant detection rates and overlap were then compared between these subgroups to evaluate whether metastatic status impacted observed differences in variant detection between the 2 assays.

### Statistical methods

Descriptive statistics were used to summarize socio-demographic and clinical characteristics and complementary testing rates were expressed as percentages. We also performed univariate and multivariate logistic regression modeling of discordance (ie, the “outcome” is a patient having a mutation detected in one test but not the other) in any of the 104 genes included in the analysis (Table S1 in the manuscript) using age at diagnosis, gender, race, ethnicity, smoking status, time between xF and xT sample collections, histology, and metastatic status as predictors.

## Results

### Patient demographics and clinical characteristics

**Figure 1:**
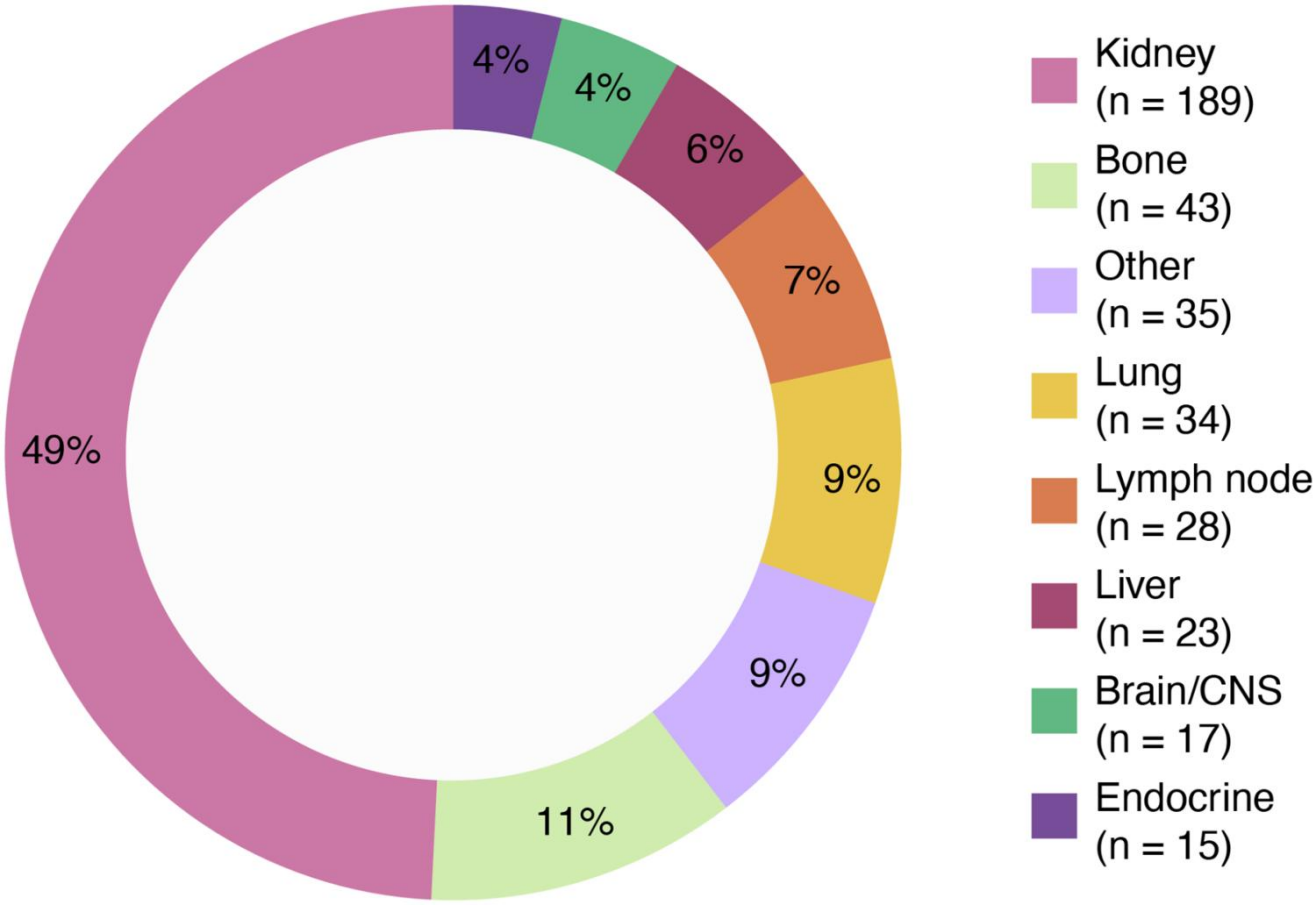
Breakdown of tissue biopsy site. Endocrine is defined as the pancreas, adrenal gland, and thyroid. Others are defined as soft tissue, urinary tract, GI tract, peritoneum, retroperitoneum, pleura, abdomen, pelvis, thorax, skin of scalp and neck, and scrotum. 8 patients had unknown status.

Among 392 patients, the median age at diagnosis was 61 years, and 71% were male. The cohort comprised a diverse population, with 75% White, 12% African-American, 4.8% Asian, and 8% from other races. The median time from tissue to blood collection was 21 days (IQR: 8, 39). Overall, 89% (*n* = 348) of patients had their tissue sample collected first. At the time of both sample collections, 66% (*n* = 259) of patients had metastatic disease. Of the metastatic patients that had both initial diagnosis date and metastatic date documented (*n* = 245), 79% of patients were de novo metastatic, defined as metastatic within 6 months of initial diagnosis. Overall, 64% were clear cell, 8.9% were papillary, 3.3% were chromophobe, and 0.3% each were collecting duct, medullary, or translocation. 23% of patients had their renal cancer histology not specified (Table 1). The most common tissue collection sites were kidney (49%, *n* = 189), bone (11%, *n* = 43), lung (9%, *n* = 34), lymph node (7%, *n* = 28), liver (6%, *n* = 23), and brain/CNS (4%, *n* = 17) (Figure 1). Most patients had not received systemic therapy prior to both specimen collection (83.7%, *n* = 324). Of the patients with documentation of treatment prior to both sample collections (17%, *n* = 68), the most frequently received treatments were ipilimumab + nivolumab (31%, *n* = 21/68), cabozantinib + nivolumab (16%, *n* = 11/68), and axitinib + pembrolizumab (16%, *n* = 11/68) (Table S3).

**Table 1:** Demographics table.

Characteristic	*N* = 392
**Age at diagnosis**	
** Median (IQR)**	61 (52, 68)
** Range**	19, 89
** Unknown**	21
**Gender**	
** Male**	280 (71%)
** Female**	112 (29%)
**Race**	
** White**	187 (75%)
** Black or African American**	31 (12%)
** Other Race**	20 (8.0%)
** Asian**	12 (4.8%)
** Unknown**	142
**Ethnicity**	
** Not Hispanic or Latino**	141 (80%)
** Hispanic or Latino**	36 (20%)
** Unknown**	215
**Smoking status**	
** Never smoker**	174 (57%)
** Current/former smoker**	133 (43%)
** Unknown**	85
**Days from tissue collection to blood collection**	
** Median (IQR)**	21 (8, 39)
** Range**	−63, 90
**Metastatic prior to earliest sample collection**	
** Yes**	259 (66%)
** Unknown**	2
**Histology group**	
** CC**	249 (64%)
** NOS**	92 (23%)
** Papillary**	35 (8.9%)
** Chromophobe**	13 (3.3%)
** Collecting duct**	1 (0.3%)
** Medullary**	1 (0.3%)
** Translocation**	1 (0.3%)

### Tissue testing

**Figure 2:**
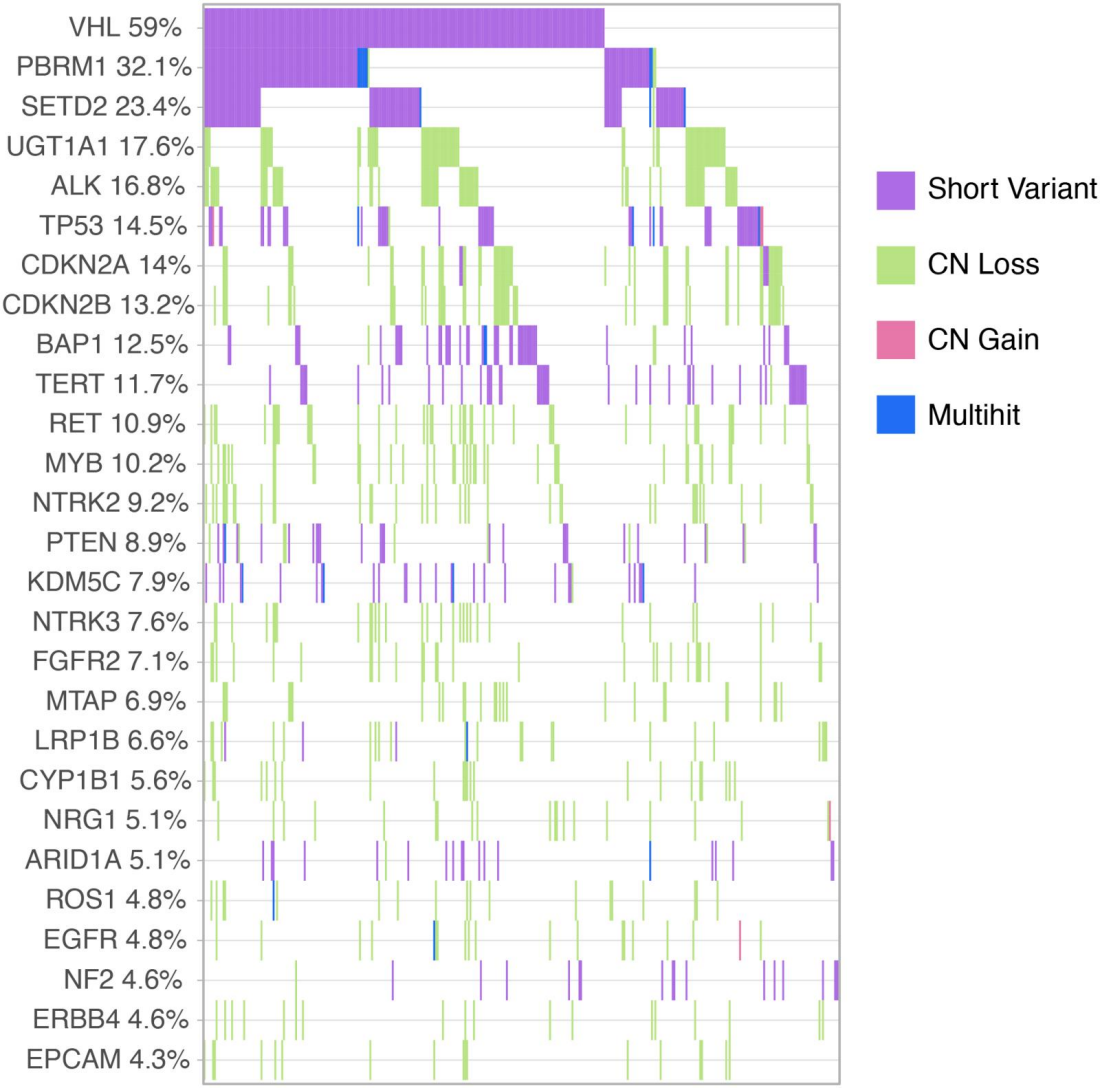
Prevalence of molecular alterations detected by solid-tissue testing. Figure restricted to genes with alterations in >=4% of population. Clinically notable alterations detected below the 4% threshold included those in the following genes: TSC1 (*n* = 14, 3.6%), BRCA2 (*n* = 13, 3.3%), MTOR (*n* = 10, 2.6%), ATM (*n* = 7, 1.8%), and PIK3CA (*n* = 7, 1.8%).

The most frequently observed genomic alterations in tissue were in *VHL* (59%), followed by *PBRM1* (32%). Other frequently altered and clinically important genes included *SETD2* (23%), *TP53* (15%), *CDKN2A* (14%), *CDKN2B* (13%), *PTEN* (8.9%) and MTAP (7.0%) (Figure 2). Additional alteration prevalences are shown and described in Figure 2. Potential germline alterations are shown in Table S4.

### ctDNA testing

**Figure 3:**
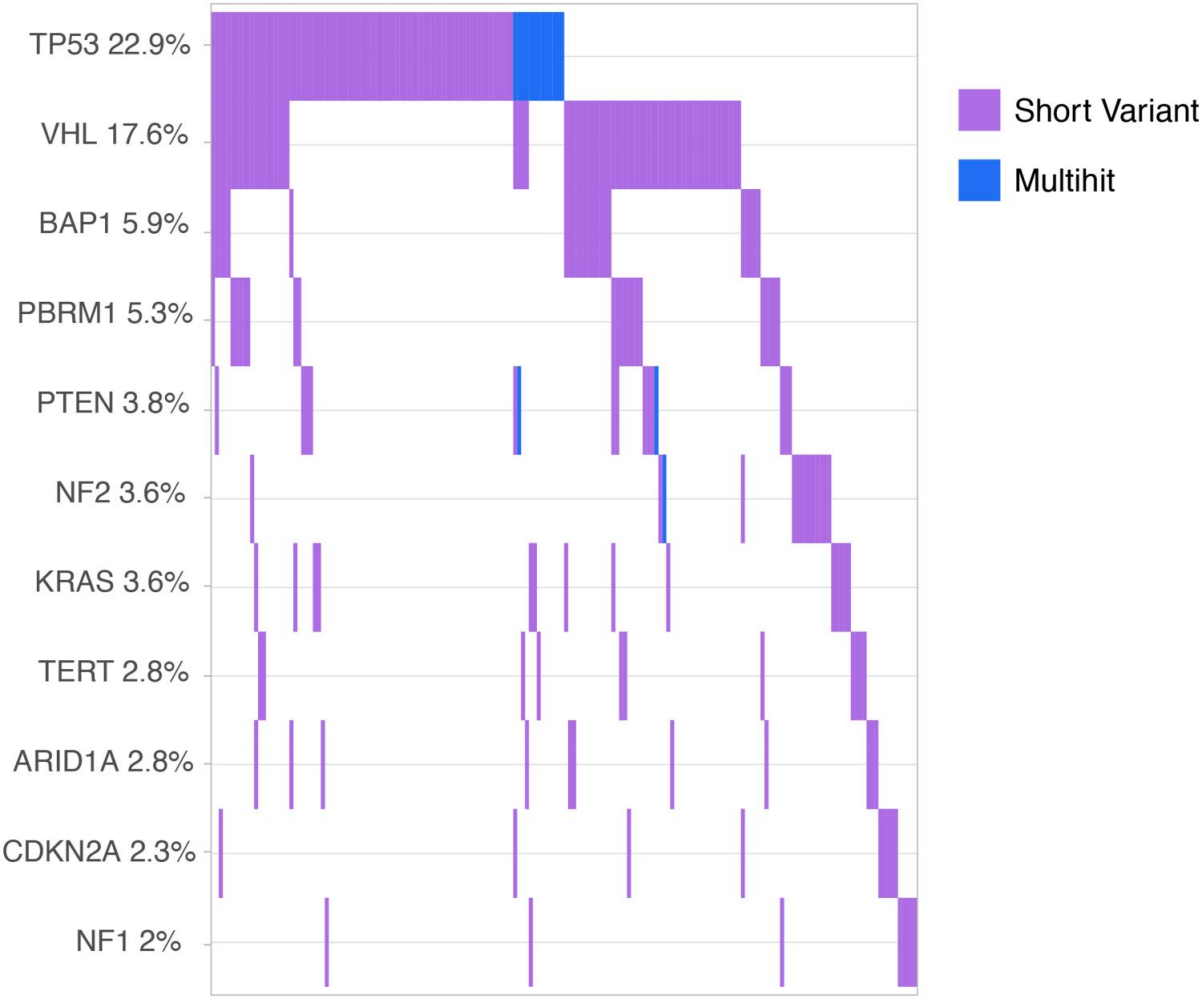
Prevalence of molecular alterations detected by ctDNA testing. Restricted to genes altered in >=2% of the population. Clinically notable alterations detected below the 2% threshold included those in the following genes: ATM (*n* = 6, 1.5%), PIK3CA (*n* = 6, 1.5%), TSC1 (*n* = 5, 1.3%), BRCA2 (*n* = 4, 1.0%), and MTOR (n = 2, 0.5%).

In our ctDNA analysis, the most frequently altered genes were TP53 (23%), VHL (18%), BAP1 (6%), and PBRM1 (5%); notably, 176 out of 392 patients did not have any pathogenic or likely pathogenic variants detected in the 104 genes analyzed. Additional notable alterations included those in *PTEN* (4%), *KRAS* (4%), and *NF2* (4%) (Figure 3).

### Differences in variant detection between solid-tissue and ctDNA testing

**Figure 4:**
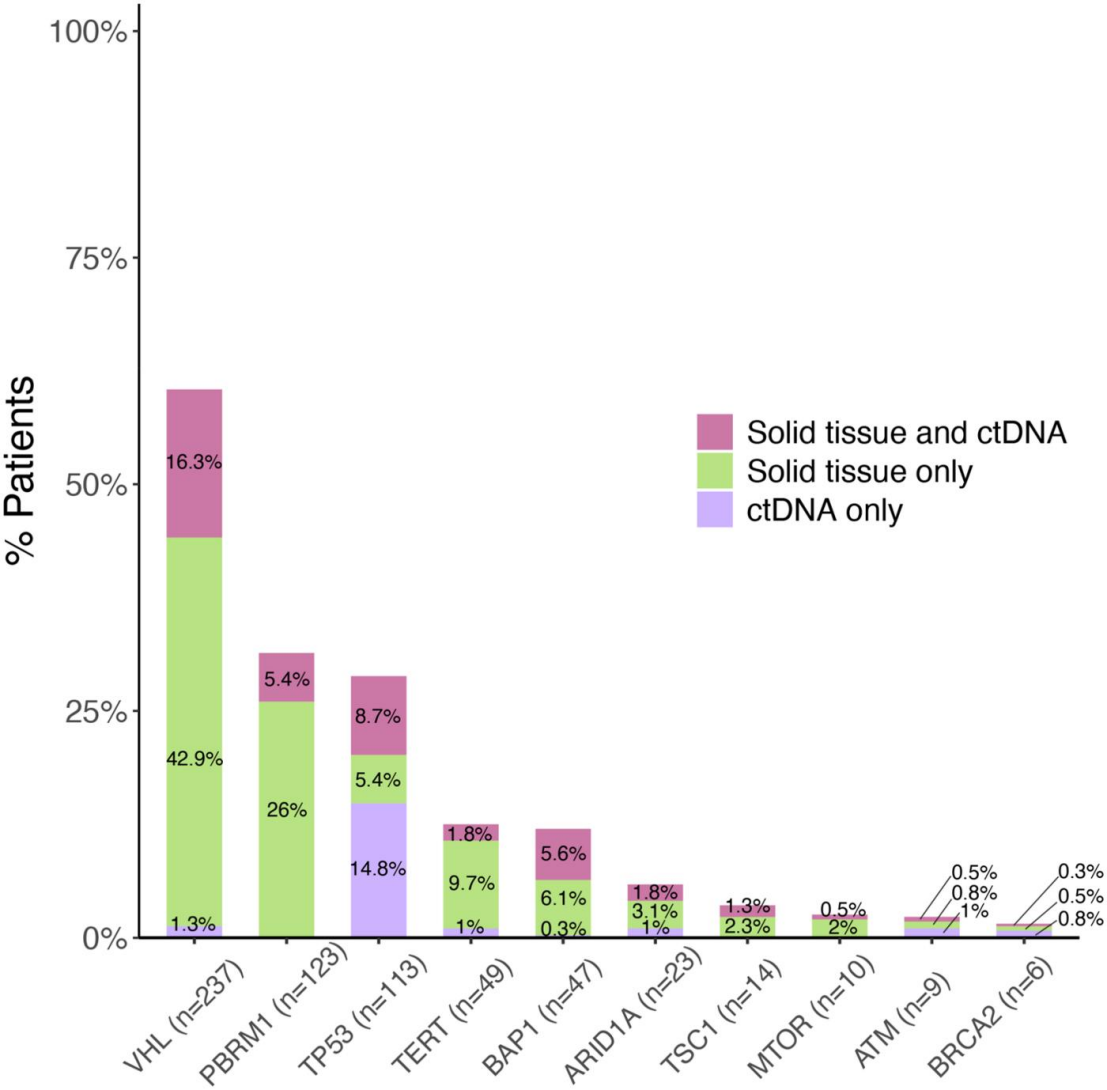
Breakdown of alterations according to assay detection type, including assay unique alterations and those detected by both assays.

The combination of tissue and ctDNA testing increased the overall detection of genomic alterations. For instance, *VHL* alterations were detected by *either* assay in 60% of patients (*n* = 237), compared with 59% that would have been observed if only solid-tissue testing (xT) was performed—an additional 5 patients had variants detected only via ctDNA testing. Conversely, all *PBRM1* variants (31%, *n* = 123) were detected by solid-tissue testing, with no patients harboring a variant uniquely detected via ctDNA testing. Combining the results of alterations found in tissue or ctDNA assays, *TP53* alterations were detected by either assay in 29% of the cohort (*n* = 113/392); using solid-tissue testing results only, the prevalence of *TP53* alterations was 14% (*n* = 55/392), but ctDNA testing revealed an additional 14.8% (*n* = 58/392 patients) with alterations in this gene. Other notable findings included alterations in the *BAP1* gene, which were detected in 12% of the tissue or ctDNA cohorts combined (*n* = 47) and only 1 patient harbored a *BAP1* alteration detected solely via ctDNA testing. Detection of alterations in *TERT, ARID1A*, *ATM*, and *BRCA2* also increased with concurrent tissue and ctDNA testing compared to tissue testing alone (Figure 4).

To assess what fraction of patients had variants detected amongst those uniquely detected in ctDNA analyses as a result of CH, we performed additional analyses to investigate these variants in the normal sample for the subset of samples with normal-tissue sequencing (see Methodology). Of the 58 patients harboring a TP53 alteration that was detected only in ctDNA, 50 had normal sequencing results allowing for us to specifically assess CH. Of these 50 samples, 5 were determined to be due to CH (10%). Thus, among the population with tumor-normal matched testing, CH was not detected in 90% of the TP53 alterations found only in ctDNA. Similar results were seen for other genes (Table S5), highlighting that CH was not detected in a large majority of variants uniquely detected via ctDNA. Median ctDNA VAF for suspected CHIP and non-CHIP alterations in select genes is available in Table S6.

**Figure 5:**
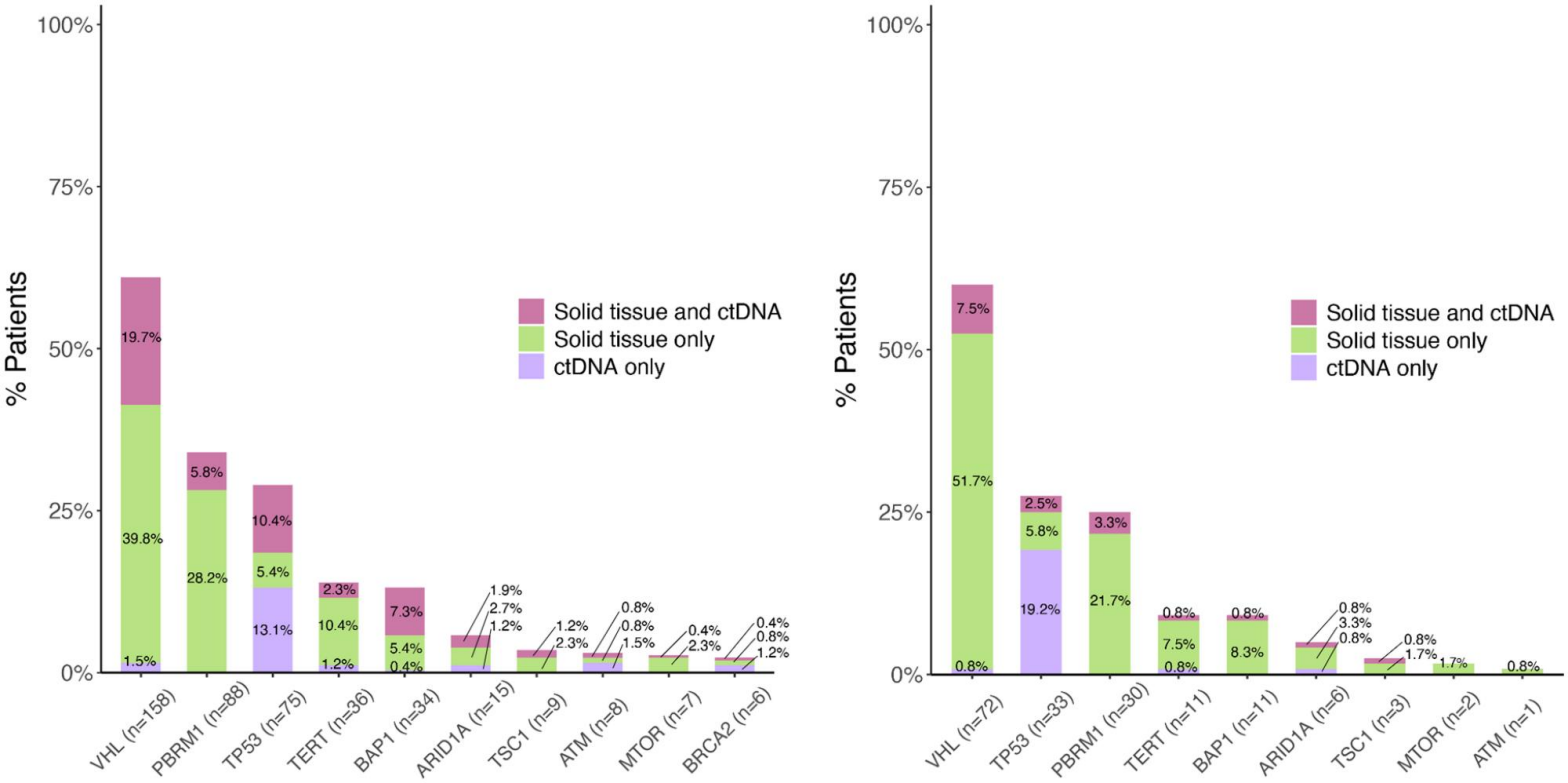
Alterations identified in solid tissue and ctDNA (stratified based on metastasis) with metastatic patients shown on the left and non-metastatic patients on the right.

We also observed that there was a higher agreement between assays when looking at somatic alterations in select genes among patients with metastatic disease compared to those without metastases. Specifically, alterations in *VHL* were detected by *both* assays for 20% of patients with metastases versus 7.5% for those without. Similar findings were observed in *PBRM1* (6% vs 3%), *TP53* (10% vs 2.5%), *TERT* (2% vs 1%), *BAP1* (7% vs 1%), *ARID1A* (2% vs 1%), and *ATM* (1% vs 0%) (Figure 5). On logistic regression modeling for discordance, time between sample collections, histology, and metastatic status were significant (*P* < .05) predictors of discordance in univariate analysis. On conducting multivariate model utilizing these variables, those patients who were metastatic prior to sample collection and NOS (Not otherwise specified) histology are less likely to have discordant findings (OR 0.87, *P* = .012 and OR 0.85, *P* =.007, respectively) (Figure S1).

## Discussion

In this study, we explored the genomic landscape of RCC using both tissue-based (Tempus xT) and ctDNA NGS (Tempus xF). The uniqueness of this dataset lies in the matched tissue and blood samples, providing a comprehensive view that is further enriched by clinical data, allowing for a more precise assessment of a tumor’s molecular profile. Our findings suggest that integrating ctDNA profiling with tissue-based NGS improves the overall detection of genomic alterations, reflecting the complementary information captured by each platform. Notably, alterations in genes such as *VHL*, *TP53*, *TERT*, *ARID1A*, *ATM*, and *BRCA2* were detected at higher rates when both methods were used together, underscoring the complementarity of ctDNA and tissue testing in the molecular profiling of RCC.

ctDNA has garnered attention for its broad utility in cancer management, with roles in identifying clinically important biomarkers, diagnosis, prognosis, and treatment monitoring across various malignancies, including RCC.^21^^,^^22^ Furthermore, tissue is not always available for NGS due to challenges associated with invasive biopsies or tissue failure during the NGS analysis. Despite the relatively low ctDNA shedding in RCC compared to other cancers, liquid assays for treatment selection, such as those that identify specific genomic biomarkers, have shown promise.^17^^,^^18^ ctDNA offers a key advantage over tissue biopsies by capturing RCC’s spatial and temporal tumor heterogeneity.^3^^,^^23^ In our cohort, pathogenic or likely pathogenic variants were identified in 216 of 392 patients (55%) across the 104 genes analyzed in the ctDNA panel, a detection rate consistent with previously published data.^24^^,^^25^ Notably, genomic alterations in VHL, TP53, TERT, BAP1, ARID1A, ATM, and BRCA2 were exclusively detected through ctDNA. This aligns with findings from a separate study of 112 matched mRCC patients, where blood-based NGS identified 46.9% of actionable alterations that were missed by tissue-based profiling.^18^

Our findings further highlight the advantage of integrating ctDNA with tissue-based NGS to provide a more complete genomic landscape of RCC. Several unique genomic alterations were detected exclusively in ctDNA, which could have potential therapeutic implications. For example, of the patients with *BRCA2* alterations (*n* = 6), 50% had unique findings in ctDNA results (Figure 4). Additionally, of the patients with ATM alterations (*n* = 9), 44% had unique findings in ctDNA results (Figure 4). Importantly, detection of alterations within the DNA damage response (DDR) pathway may identify novel therapeutic options and help with clinical trial considerations. Indeed, there is growing evidence supporting the role of PARPi in RCC, particularly in DDR-deficient neoplasms like those with BAP1 and other altcanerations.^26^^,^^27^ Ongoing clinical trials are exploring the therapeutic potential of PARPi in these settings with an interim analysis from Phase II indicating some clinical activity of PARPi as single agent in patients with BAP1 mutations.^27–29^ Together, these findings highlight the complementary role of ctDNA in identifying potentially actionable genomic alterations that may be missed by tissue biopsy alone due to spatial sampling bias or tumor evolution. As the therapeutic landscape continues to expand with novel targeted agents and precision-medicine driven clinical trials, ctDNA-only findings may offer clinically meaningful information to support treatment decision-making, at the discretion of the treating physician.

We also found a higher agreement between tissue and ctDNA testing in patients with metastatic RCC, especially for alterations in genes such as *VHL, TP53*, and *BAP1*. This suggests that ctDNA profiling may have increased sensitivity in detecting alterations in metastatic RCC, likely due to higher tumor burden and greater ctDNA shedding in these patients, as supported by previous studies.^13^^,^^23^ For instance, in a smaller study of ctDNA-positive metastatic RCC patients (*n* = 14), Bacon et al. reported a tissue–ctDNA concordance rate of 77%, with VHL, PBRM1, and BAP1 being the most frequently altered genes.^17^ In contrast, in our study only moderate numbers of variants were detected in both solid-tissue and ctDNA analyses (Figure 4, magenta bars), with less than 50% of the variants for any individual gene. We hypothesize that these differences are driven by variations in patient populations, sequencing panels, and analytic approaches, most notably the restriction in the prior study to patients with ctDNA levels above a predefined detection threshold. This also highlights that future studies may need to incorporate more sensitive assays, such as minimal residual disease (MRD) assays, to identify trace amounts of ctDNA in the early-stage setting.

Although there are clear advantages, there are limitations to consider. First, the retrospective design and reliance on a single multimodal database limit the generalizability of our findings. Additionally, the detection of ctDNA is highly dependent on tumor burden, with lower shedding in early-stage or non-metastatic RCC cases leading to potential false negatives, highlighting the need for further analysis with MRD assays in RCC.^23^ Consistent with these previous studies, the concordance rates in non-metastatic RCC were significantly lower compared to metastatic RCC, with alterations in genes like VHL and TP53 showing reduced detection by ctDNA in non-metastatic samples. Furthermore, confounding variables such as clonal hematopoiesis (CH) may contribute to the unique variants identified in ctDNA that were not detected in tissue. The higher prevalence of ATM mutation in our ctDNA cohort could be secondary to CH variants. Several factors can cause discordant alteration findings between genomic assays. The discordant findings could stem from biological factors such as tumor heterogeneity and rates of tumor shedding. However, other technological factors across the assays such as sensitivity and genomic coverage in genes such as PBRM1 may have also contributed. Future analysis should seek to identify the prevalence of CH variants in patients with RCC as the findings will enhance the accuracy of tumor-derived somatic variant calls and may have therapeutic and risk management implications.

Despite study limitations, our analysis highlights the potential value of ctDNA testing in metastatic RCC, where it may offer insights into tumor heterogeneity, tumor evolution, and treatment response. Our findings suggest that ctDNA could serve as a complementary tool to tissue biopsies, enhancing the detection of actionable alterations and providing a real-time perspective on tumor dynamics. Given the evolving treatment paradigm that includes ICIs and TKIs, prospective trials are warranted to better understand the prognostic and predictive value of ctDNA genomic alterations and ctDNA fraction in both the early and late stage RCC settings.

## Conclusion

The analysis conducted in this study highlights the complementary nature of ctDNA profiling alongside tissue-based NGS in RCC, demonstrating an increased detection of alterations when both assays are used in tandem. The unique alterations identified through ctDNA in genes such as *BAP1*, *BRCA2*, *TP53,* and *ATM*, may have prognostic and therapeutic implications. Additionally, the higher agreement between ctDNA and tissue profiling in individuals with metastatic disease suggests the potential utility of ctDNA analysis in advanced stages of RCC. Further research is warranted to elucidate how longitudinal ctDNA analysis can delineate biomarkers of response and resistance at both the mutation and ctDNA fraction levels. Understanding these dynamics could offer valuable insights into disease progression and guide personalized treatment strategies for RCC patients.

## Supplementary Material

oyag123_Supplementary_Data

## Data Availability

The data underlying this article were provided by Tempus by permission. Data will be shared on request to the corresponding author with permission of Tempus.
