# Supplementary material for: Concurrent tissue and circulating tumor DNA analysis in renal cell carcinoma: insights from a multimodal database
Source: Oncologist. 2026 Apr 3;31(5):oyag123. doi: 10.1093/oncolo/oyag123 (PMC13127420; doi:10.1093/oncolo/oyag123)
Supplement: oyag123_Supplementary_Data [file oyag123_supplementary_data.docx]

**Supplementary Table 1: Listing of the 104 genes assessed in this study.**

| AKT1 | DPYD | KIT | PDGFRB |
| --- | --- | --- | --- |
| AKT2 | EGFR | KMT2A | PIK3CA |
| ALK | ERBB2 | KRAS | PIK3R1 |
| APC | ERRFI1 | MAP2K1 | PMS2 |
| AR | ESR1 | MAP2K2 | PTCH1 |
| ARAF | EZH2 | MAPK1 | PTEN |
| ARID1A | FBXW7 | MET | PTPN11 |
| ATM | FGFR1 | MLH1 | RAD51C |
| ATR | FGFR2 | MPL | RAF1 |
| B2M | FGFR3 | MSH2 | RB1 |
| BAP1 | FGFR4 | MSH3 | RET |
| BRAF | FLT3 | MSH6 | RHOA |
| BRCA1 | FOXL2 | MTOR | RIT1 |
| BRCA2 | GATA3 | MYC | RNF43 |
| BTK | GNA11 | MYCN | ROS1 |
| CCND1 | GNAQ | NF1 | SDHA |
| CCND2 | GNAS | NF2 | SMAD4 |
| CCND3 | HNF1A | NFE2L2 | SMO |
| CCNE1 | HRAS | NOTCH1 | SPOP |
| CD274 | IDH1 | NPM1 | STK11 |
| CDH1 | IDH2 | NRAS | TERT |
| CDK4 | JAK1 | NTRK1 | TP53 |
| CDK6 | JAK2 | PALB2 | TSC1 |
| CDKN2A | JAK3 | PBRM1 | TSC2 |
| CTNNB1 | KDR | PDCD1LG2 | UGT1A1 |
| DDR2 | KEAP1 | PDGFRA | VHL |
|  |  |  |  |

**Supplementary Table 2: Listing of the 63 genes assessed for germline variants.**

| APC | KIT | RAD51C |
| --- | --- | --- |
| ATM | MAX | RAD51D |
| AXIN2 | MEN1 | RB1 |
| BAP1 | MET | RET |
| BARD1 | MLH1 | RUNX1 |
| BMPR1A | MSH2 | SDHA |
| BRCA1 | MSH3 | SDHAF2 |
| BRCA2 | MSH6 | SDHB |
| BRIP1 | MUTYH | SDHC |
| CDH1 | NF1 | SDHD |
| CDK4 | NF2 | SMAD4 |
| CDKN2A | NTHL1 | SMARCA4 |
| CEBPA | PALB2 | SMARCB1 |
| CHEK2 | PDGFRA | STK11 |
| DICER1 | PHOX2B | SUFU |
| EGFR | PMS2 | TMEM127 |
| EPCAM | POLD1 | TP53 |
| ETV6 | POLE | TSC1 |
| FH | PRKAR1A | TSC2 |
| FLCN | PTCH1 | VHL |
| GATA2 | PTEN | WT1 |
|  |  |  |

**Supplementary Table 3: Treatments received in the population of patients that had documentation of treatment prior to xT and xF sample collection.**

| **Agent** | **N = 68** |
| --- | --- |
| **ipilimumab + nivolumab** | 21 (31%) |
| **cabozantinib + nivolumab** | 11 (16%) |
| **axitinib + pembrolizumab** | 11 (16%) |
| **pembrolizumab** | 10 (15%) |
| **nivolumab** | 10 (15%) |
| **pazopanib** | 8 (12%) |
| **cabozantinib** | 7 (10%) |
| **sunitinib** | 6 (8.8%) |
| **everolimus + lenvatinib** | 3 (4.4%) |
| **interleukin-2** | 3 (4.4%) |
| **lenvatinib + pembrolizumab** | 3 (4.4%) |
| **axitinib** | 2 (2.9%) |
| **belzutifan** | 2 (2.9%) |
| **tivozanib** | 2 (2.9%) |
| **octreotide** | 1 (1.5%) |
| **investigational new drug + nivolumab** | 1 (1.5%) |
| **sorafenib** | 1 (1.5%) |
| **temsirolimus** | 1 (1.5%) |
| **denosumab + pazopanib** | 1 (1.5%) |
| **atezolizumab + interferon alfa-2b** | 1 (1.5%) |
| **atezolizumab + bevacizumab + interferon alfa-2a** | 1 (1.5%) |
| **cabozantinib + ipilimumab + nivolumab** | 1 (1.5%) |
| **atezolizumab + bevacizumab + investigational new drug** | 1 (1.5%) |
| **ipilimumab + nivolumab + pembrolizumab** | 1 (1.5%) |
| **cisplatin + gemcitabine** | 1 (1.5%) |
| **everolimus** | 1 (1.5%) |
| **axitinib + gemcitabine** | 1 (1.5%) |
| **trastuzumab** | 1 (1.5%) |

a

**Supplementary Table 4: Potential germline alterations identified in cohort. Only patients with tumor-normal testing were included (n=355).**

| **Gene** | **N = 355** |
| --- | --- |
| **CHEK2** | 8 (2.3%) |
| **MUTYH** | 4 (1.1%) |
| **ATM** | 2 (0.6%) |
| **BRIP1** | 2 (0.6%) |
| **BRCA2** | 1 (0.3%) |
| **FH** | 1 (0.3%) |
| **FLCN** | 1 (0.3%) |
| **MSH3** | 1 (0.3%) |
| **PMS2** | 1 (0.3%) |
| **RAD51C** | 1 (0.3%) |
| **RB1** | 1 (0.3%) |

**Supplementary Table 5: Breakdown of variants detected only in ctDNA according to CH status.**

|  | Patients with ctDNA-unique variants detected in T/N matched cohort (n=355) | Number of patients with CH or likely CH variants (n=23) | Fraction of patients with ctDNA-unique variants that are CH or likely CH |
| --- | --- | --- | --- |
| VHL | 4 | 0 | 0 |
| PBRM1 | 0 | 0 | 0 |
| TP53 | 50 | 5 | 0.1 |
| TERT | 4 | 1 | 0.25 |
| BAP1 | 1 | 0 | 0 |
| ARID1A | 3 | 1 | 0.33 |
| TSC1 | 0 | 0 | 0 |
| MTOR | 0 | 0 | 0 |
| ATM | 4 | 1 | 0.25 |
| BRCA2 | 3 | 1 | 0.33 |

Supplementary Table 6: Median ctDNA VAF in Suspected CHIP vs Non-CHIP Mutations by Gene

| **Gene** | **Median ctDNA VAF in suspected CHIP population (min, max)** | **Median ctDNA VAF in non-CHIP population (min, max)** |
| --- | --- | --- |
| TP53 | 2.09 (0.48 - 12.06) | 0.35 (0.06 - 40.79) |
| TERT | 13.91 (13.91 - 13.91) | 1.86 (0.21 - 22.53) |
| BRCA2 | 0.54 (0.54 - 0.54) | 24.51 (0.7 - 48.32) |
| ATM | 3.51 (3.51 - 3.51) | 1.51 (0.66 - 49.04) |
| ARID1A | 1.74 (1.74 - 1.74) | 2.27 (0.89 - 16.95) |
| VHL | NA (NA - NA) | 1.67 (0.11 - 52.58) |
| PBRM1 | NA (NA - NA) | 2.15 (0.25 - 14.65) |
| BAP1 | NA (NA - NA) | 2.46 (0.24 - 21.53) |
| TSC1 | NA (NA - NA) | 9.75 (3.78 - 12.29) |
| MTOR | NA (NA - NA) | 0.8 (0.79 - 0.8) |

Supplementary figure 1: Multivariate logistic regression evaluating predictors of discordant tissue and ctDNA findings


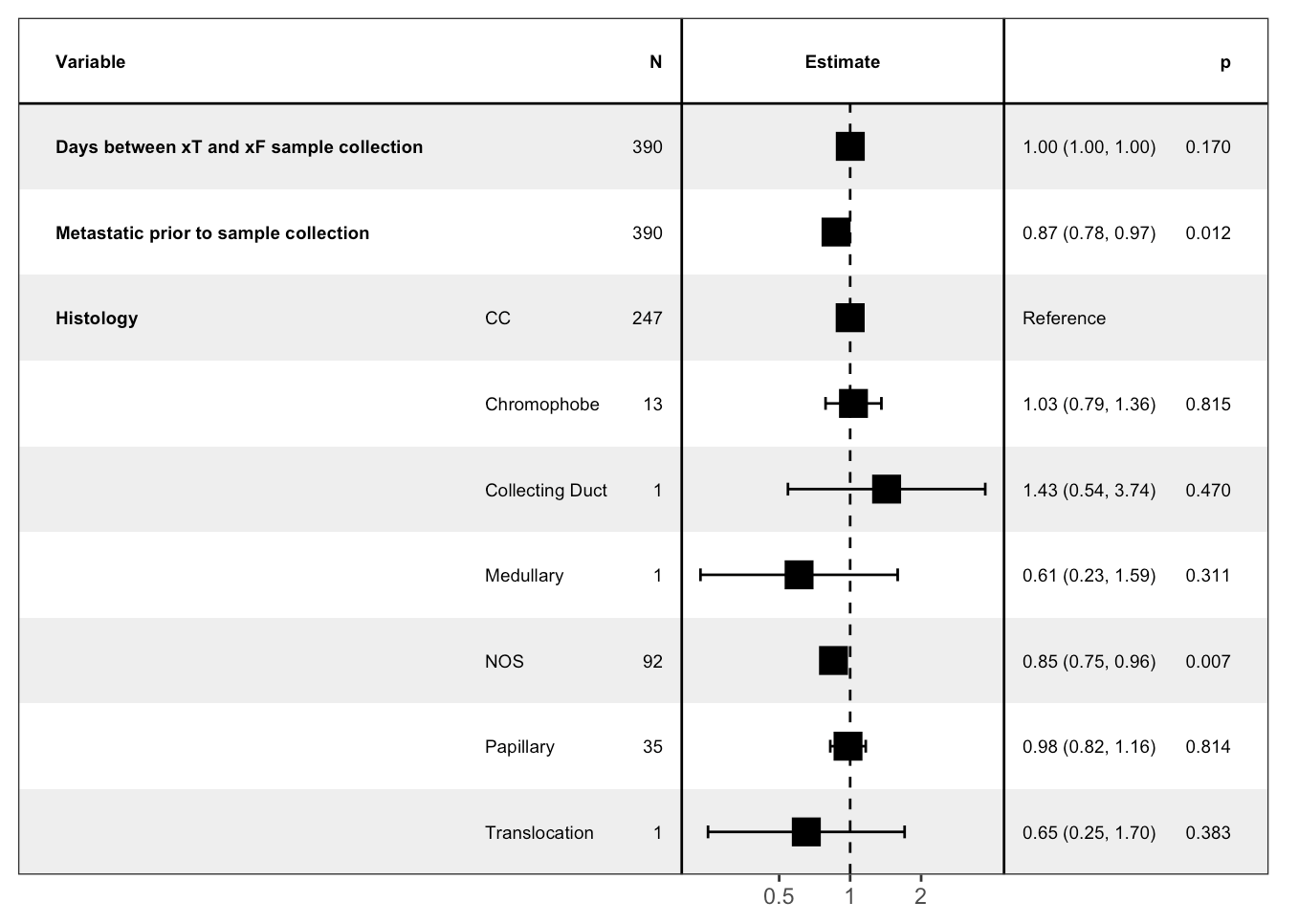


Variables included in the multivariable logistic regression model were selected based on significance in univariate analysis and included time between tissue and ctDNA sample collection, histologic subtype, and metastatic status at the time of sampling. xT :Tissue Assay, xF :ctDNA assay), CC : Clear Cell, NOS: Not otherwise specified.
